# Passive Repetitive Stretching for a Short Duration within a Week Increases Myogenic Regulatory Factors and Myosin Heavy Chain mRNA in Rats' Skeletal Muscles

**DOI:** 10.1155/2013/493656

**Published:** 2013-05-23

**Authors:** Yurie Kamikawa, Satoshi Ikeda, Katsuhiro Harada, Akihiko Ohwatashi, Akira Yoshida

**Affiliations:** Department of Rehabilitation and Physical Medicine, Graduate School of Medical and Dental Sciences, Kagoshima University, Kagoshima, 8-35-1 Sakuragaoka, Kagoshima-shi, Kagoshima 890-8520, Japan

## Abstract

Stretching is a stimulation of muscle growth. Stretching for hours or days has an effect on muscle hypertrophy. However, differences of continuous stretching and repetitive stretching to affect muscle growth are not well known. To clarify the difference of continuous and repetitive stretching within a short duration, we investigated the gene expression of muscle-related genes on stretched skeletal muscles. We used 8-week-old male Wistar rats (*N* = 28) for this study. Animals medial gastrocnemius muscle was stretched continuously or repetitively for 15 min daily and 4 times/week under anesthesia. After stretching, muscles were removed and total RNA was extracted. Then, reverse transcriptional quantitative real-time PCR was done to evaluate the mRNA expression of MyoD, myogenin, and embryonic myosin heavy chain (MyHC). Muscles, either stretched continuously or repetitively, increased mRNA expression of MyoD, myogenin, and embryonic MyHC more than unstretched muscles. Notably, repetitive stretching resulted in more substantial effects on embryonic MyHC gene expression than continuous stretching. In conclusion, passive stretching for a short duration within a week is effective in increasing myogenic factor expression, and repetitive stretching had more effects than continuous stretching for skeletal muscle on muscle growth. These findings are applicable in clinical muscle-strengthening therapy.

## 1. Introduction

Muscle weakness is the cause of numerous disabilities in daily life—a problem often encountered in rehabilitation medicine. Several studies have attempted to improve the muscle strength by techniques such as voluntary contraction [1], electrical stimulation, or a combination of the two [2]. Numerous studies have shown that mechanical stimulation promotes gene expression and protein accumulation in muscles [3]. Passive stretching, a type of mechanical stimulation, has been suggested as an effective method for inducing muscle hypertrophy [3]. Passive stretching is performed in rehabilitation medicine worldwide, and it is simple to apply for preventing muscle weakness and shortening muscle length as well as maintaining the range of joints [4–6]. Passive stretching induces muscles growth toward longitudinal and parallel directions [6]. According to previous reports, a 30-min bout of passive stretching induces the expression of myogenic differentiation factor in soleus muscles [4]. once stretches more than 1 hour [7]. Many reports have shown the benefits of continuous stretching by taping or casting, although these techniques are not commonly used in rehabilitation medicine.

Cyclic stretching *in vitro* induces the differentiation of mouse myoblast C2C12 cells and the activation of satellite cells [8, 9]. By contrast, few studies have described the effect of passive repetitive stretching *in vivo*, which is used frequently in clinical rehabilitation settings [7, 10, 11]. Furthermore, mechanical simulation has been described as a regulator of gene expression in numerous studies [12–14]. However, the differences between continuous and repetitive stretching in the skeletal muscle have not been investigated *in vivo.*

The pathways of muscle hypertrophy include several steps. The myogenic regulatory pathway is a part of the process of muscle hypertrophy consisting of 3 steps: differentiation, fusion, and maturation [15]. Myogenic differentiation 1 (MyoD) and myogenin are members of the myogenic regulatory factor (MRF) family. MyoD is a key regulator of the first step of the hypertrophic pathway (differentiation), while myogenin functions in the second step of the pathway (fusion). MyoD and myogenin are both downregulated by SmaD3 and myostatin expression [16]. Additionally, mammalian skeletal muscle consists of 9 myosin heavy chain (MyHC) isoforms. Embryonic and neonatal MyHC isoforms are typically expressed during the development stage, but they are absent in normal adult rat muscles [17, 18]. Expression of embryonic MyHC in an adult rat muscle is apparent in myotubes which consist of activated satellite cells during the process of regeneration [19]. Passive stretching alters the composition of myosin, consisting of IIA MHC [20]. 

In this study, we investigated the differences in the effects of continuous versus repetitive passive stretching, applied for a short duration (at several days during a 1-week period) to medial gastrocnemius muscles in rats. We evaluated the expression of MyoD, myogenin, and embryonic MyHC genes by using molecular biological techniques.

## 2. Materials and Methods

### 2.1. Animals and Experimental Groups

We used 8-week-old male Wistar rats (*N* = 28) for this study. The study protocol was carried out in accordance with the Guide for Animal Experimentation of the faculty in the Department of Medicine of Kagoshima University and the guidelines of the US National Institute of Health, and this study was approved by the animal experiment committee of Kagoshima University. These animals were housed in plastic cages in an environmentally controlled room with a 12/12-hour light-dark cycle ad libitum.

The rats were anesthetized with sodium pentobarbital (40 mg/kg) in the peritoneal pathway and divided into 3 groups. In the first group of rats (*n* = 11), the right gastrocnemius muscles were stretched continuously by manual ankle dorsiflexion for 15 min daily or 4 times a week. In the second group of rats (*n* = 11), their muscles were stretched repetitively 15 times per min for 15 min daily or 4 times a week during the same period. Contralateral muscles without stretching were also examined on the unstretched side. In the third group of rats (*n* = 6), their muscles were not stretched on either side of their legs, functioning as a control group. Twenty-four hours after the final stretch session, both sides of the medial gastrocnemius muscle were removed from the rats under deep anesthesia. The rats were then sacrificed by a lethal dose injection of sodium pentobarbital. The extracted muscles were immediately preserved in liquid nitrogen and stored at −80°C for RNA extraction.

### 2.2. RNA Isolation and Analysis

The tissues were homogenized using a hand homogenizer with Trizol reagent (10 mL/mg tissue; Invitrogen, Carlsbad, CA, USA) followed by the addition of chloroform (0.2x volume of tissue). Total RNA remained after extraction in the supernatant, and after the removal of protein and deoxyribonucleic acid by using a precipitator. Total RNA was estimated spectrophotometrically at the wavelength of 260 nm, divided into each 10 ng. We confirmed the purity of the RNA and identified the 18S and 28S ribosomal bands stained with ethidium bromide under ultraviolet light.

### 2.3. cDNA Synthesis

Ten nanograms of mRNA was washed twice with 75% ethanol and dissolved in 50 *μ*L DEPC-water (0.2 *μ*g cDNA/*μ*L). The cDNA synthesis mixture consisted of 1 ng mRNA, 2.5 *μ*M (*μ*L) Oligo-dT, and 7 *μ*L DEPC water: the total quantity was 13 *μ*L. The mixture was incubated at 65°C for 10 min and immediately cooled on ice. Secondly, 20 U (40 U/*μ*L) of dNTP mix, 3 mM MgCl_2_, 20 U (40 U/*μ*L) protector RNase inhibitor, and 10 U transcriptor reverse transcriptase were added to the incubated mixture: the total quantity was 20 *μ*L. Every round of cDNA synthesis was completed after it was incubated at 60°C for 30 min.

### 2.4. Oligonucleotide Primers

Oligonucleotide primers were designed for MyoD, myogenin, embryonic MyHC, and GAPDH as described in previous studies, using Custom Primer Software (Invitrogen, USA). 

The sequences used were derived from the following genes: rat MyoD (forward: GGAGACAATCCTCAAGCGATGC; reverse: AGCACCTGGTAAATCGGATTGG); rat myogenin (forward: ACTACCCACCGTCCATTCAC; reverse: TCGGGGCACTCACTGTCTCT); rat embryonic MyHC (forward: GAGGATCAGAGAGCTAGAGTT; reverse: ATTAAGCAGGATGGTCAGGAGCC); rat GAPDH (forward: TGGTGAAGGTCGGTGTGAAC; reverse: AGGGGTCGTTGATGGCAACA).

### 2.5. Analysis by Quantitative Real-Time Polymerase Chain Reaction

The polymerase chain reaction procedure was employed using a Light Cycler (Roche Diagnostics, Indianapolis, IN, USA) and performed with the following steps: 5 min at 95°C followed by 45 cycles of 10 s at 95°C, 5 s at 58°C, and 10 s at 72°C. After these steps were completed, the melting temperature was examined. The success of each reaction was measured based on observing the yield of a single reaction product on an agarose gel and a single peak on the DNA melting temperature curve determined at the conclusion of the reaction. The quantity of DNA was analyzed by detecting the fluorescent dye SYBR green at the point of extension. The ratio of mRNA of MyoD, myogenin, and embryonic MyHC to GAPDH was used as an internal control and compared within each group.

### 2.6. Statistics

All experiments were expressed by a mean ± standard error (SE). The Wilcoxon signed rank test was used to compare data between the stretched right muscles and unstretched left muscles of the same animals within each group. The other was examined by using one-way analysis of variance (ANOVA). A significant difference was set at a value of less than 5% (*P* < 0.05).

## 3. Results

There were no macrofindings of muscle damage. No significant differences were observed in the weight of whole gastrocnemius muscle among any of the groups evaluated (Table 1).

**Figure 1:**
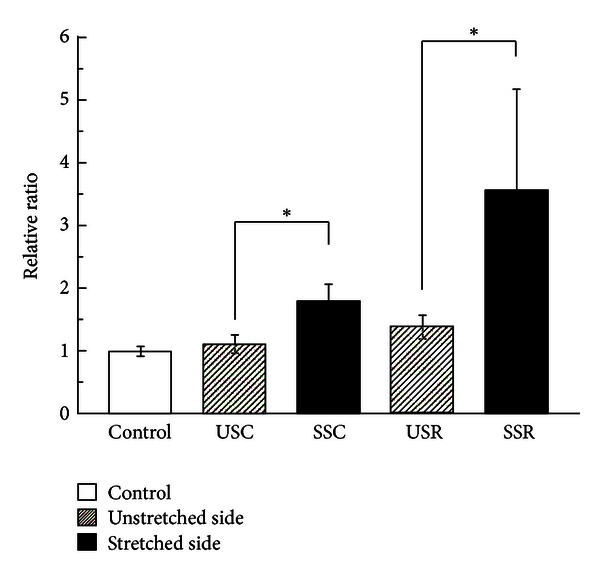
MyoD mRNA expression. Relative ratio of MyoD mRNA expression induced under different stretching conditions (mean ± SE). The open bar shows the control group; shaded bars denote the unstretched side of continuous stretching and repetitive stretching; and filled bars denote the stretched side of continuous stretching and repetitive stretching. Significant differences were displayed (*P < 0.05).

MyoD gene expression increased 1.6-fold in the continuous stretched side and 2.5-fold in the repetitive stretched side compared to each respective unstretched side, both in a statistically significant manner (*P* < 0.05). The repetitive stretching group increased 3.6-fold compared to the control group and 2.0-fold compared to the continuous stretching group; the latter increased 1.8-fold compared to the control group (Figure 1). None of the differences among the 3 groups were statistically significant.

**Figure 2:**
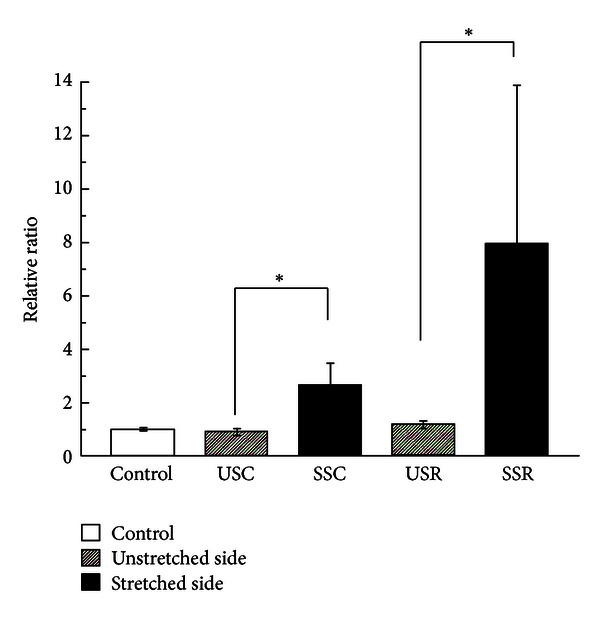
Myogenin mRNA expression. Relative ratio of myogenin mRNA expression induced under different stretching conditions (mean ± SE). The open bar shows the control group; shaded bars denote the unstretched side of continuous stretching and repetitive stretching; and filled bars denote the stretched side of continuous stretching and repetitive stretching. Significant differences were displayed (*P < 0.05).

Myogenin mRNA increased 2.9-fold in the continuous stretched side and 6.8-fold in the repetitive stretched side compared to each respective unstretched side in a statistically significant manner (*P* < 0.05). The repetitive stretched muscles increased 8.0-fold compared to the control group and 3.0-fold compared to the continuous stretching group; the latter increased 2.7-fold compared to the control group (Figure 2). None of the differences among the 3 groups were statistically significant.

**Figure 3:**
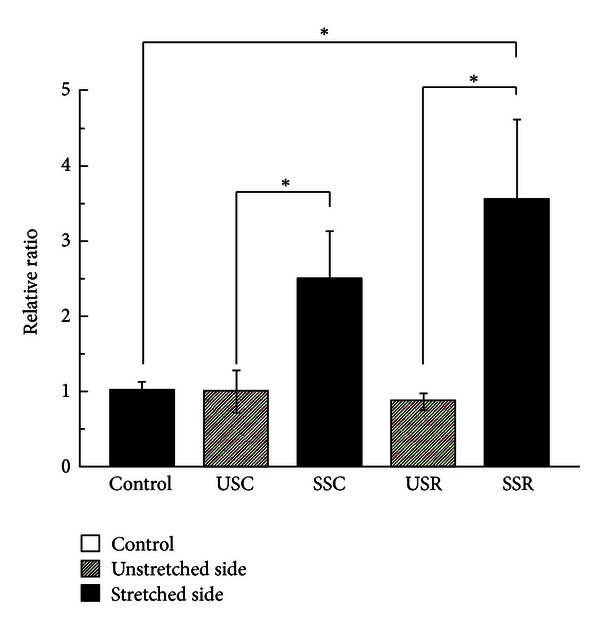
MyHC mRNA expression. Relative ratio of MyHC mRNA expression induced under different stretching conditions (mean ± SE). The open bar shows the control group; shaded bars denote the unstretched side of continuous stretching and repetitive stretching; and filled bars denote the stretched side of continuous stretching and repetitive stretching. Significant differences were displayed (*P < 0.05).

Embryonic MyHC gene expression increased 2.5-fold in the continuous stretched side and 4.1-fold in the repetitive side compared to the unstretched side, both in a statistically significant manner (*P* < 0.05). The continuous stretched muscles increased 2.5-fold compared to the control group, and the repetitive stretched muscles increased 1.4-fold compared to the continuous stretched muscles (Figure 3). Significant differences were observed between the control group and the repetitive stretched group, but not the continuous stretching group.

## 4. Discussion

Previous studies have shown that passive stretching induced muscle hypertrophy, while stretching for less than 30 min does not induce myogenesis [4, 21, 22]. In this study, we report that passive stretching for 15 min daily within a 1-week period induces the expression of MyoD, myogenin, and embryonic MyHC mRNA. Stretching for 15 min daily or [22] for several days within a week induced skeletal muscle-specific gene expression. Moreover, repetitive stretching is more effective than continuous stretching in inducing myogenesis. We observed a statistically significant difference in the expression of embryonic MyHC in the repetitive stretching group compared to the continuous stretching group, whereas no differences were observed in the expression of MyoD or myogenin. Embryonic MyHC is generated in response to the muscle developmental stage [23] as well as the stage of new muscle fiber formation in adults [24, 25]. Therefore, repetitive stretching has greater effects than continuous stretching on muscle fiber formation.

Our results indicated that myogenin and MyoD mRNA expression were significantly increased in continuous and repetitive stretching muscles compared to unstretched contralateral muscles. Peviani et al. reported that daily stretching sessions within 15 min of a passive stretch did not increase MyoD mRNA expression in soleus muscles [11]. Additionally, differential expression patterns of MRF mRNA in stretching were observed between plantaris and soleus muscles [26]. There is also the possibility of a differential response in stretching between fast and slow muscles.

A significant increase in embryonic MyHC expression was observed in repetitive stretching compared with continuous stretching. Passive stretching activates the integrin-mediated signaling pathway which transforms mechanical to biochemical stimuli. Mechanotransduction plays an important role in the integrin-mediated signaling pathway *in vivo*. Integrin consists of 16 alpha and 8 beta subunits. The beta-1D integrin subunit and nitric oxide play pivotal roles in skeletal muscles [27]. 

Mechanical stimuli induce the production of mechanogrowth factor, a splicing variant of insulin-like growth factor, which induces the differentiation of mesodermal progenitors into mature myotubes. Mechanical stretching alone could induce the differentiation of myoblasts into mature myotubes *in vitro* [26]. 

Intermittent stretching induces osteoblast-like cells to become hypertrophic [28]. Moreover, cyclic stretching augments the effect of static stretching, whereas a solitary static stretch induces muscle hypertrophy [29]. Repetitive stretching continually affected the conformation change of the extracellular matrix, whereas continuous stretching affected the mechanical stimulation of the matrix at a single time. In our study, repetitive stretching was more effective than continuous stretching, functioning in a cumulative manner.

Previous studies have demonstrated the effects of continuous stretching on the skeletal muscle by taping or casting to maintain muscles in a stretched state [4, 22]. However, casting induces articular contracture—an effect that is avoided with the repetitive stretching of muscles. Passive stretching in combination with electrical stimulation accelerates muscle hypertrophy more effectively than either technique alone, thereby increasing MRF expression [30]. 

Several studies have reported that passive stretching induces skeletal muscle hypertrophy [7, 10]. By contrast, cyclic mechanical straining inhibits skeletal myogenesis in C2C12 cells [31]. However, since cyclic stretching induced the differentiation of satellite cells, it suppressed differentiated satellite cells from forming myotubes [32]. Finally, the stretching of irradiated skeletal muscles in the absence of satellite cells induced muscle hypertrophy [2]. 

 Hypertrophy of the skeletal muscle does not have to exist in the satellite cells. Therefore, repetitive stretching, as performed in our study, may induce the effects of muscle hypertrophy.

We stretched medial gastrocnemius muscles in rats under deep anesthesia without applying resistant force within the natural range of motion in order to avoid muscle damage. Passive stretching for 15 min daily within a 1-week period induced the expression of MyoD, myogenin, and embryonic MyHC mRNA. We examined the potential of remote hormonal factor participation. No significant differences were observed between continuous and repetitive unstretched sides, and the control groups showed stretch effects that were restricted to the affected region. This observation demonstrates that remote hormonal factors did not influence our results.

This study highlights the advantages of passive stretching for short durations daily. From the clinical view, additional evidence is required to confirm that passive stretching once daily for a short duration during long periods induces muscle hypertrophy. Additional studies are required to determine the effects of passive stretching applied for longer periods.

In conclusion, passive stretching for a short duration once daily at several days within a 1-week period is effective in the growth of the skeletal muscle. Repetitive stretching is suggested to have greater effects than continuous stretching. These findings suggest that passive stretching is useful in the prevention and maintenance of skeletal muscle tone in patients who are unconscious or paralyzed.

## Figures and Tables

**Table 1:** Body weight and muscle weight.

Groups	Increase in body weight (g)	Muscle weight (mg)
Control	26.67 ± 2.11	1710.0 ± 18.5
Continuous Stretch	33.50 ± 6.26	
Unstretched		1893.2 ± 80.8
Stretched		1847.4 ± 79.8
Repetitive Stretch	30.50 ± 5.14	
Unstretched		1881.0 ± 61.4
Stretched		1858.8 ± 63.2

Mean ± standard error.
